# Cerebellar Purkinje cell firing promotes conscious recovery from anesthesia state through coordinating neuronal communications with motor cortex

**DOI:** 10.7150/thno.89592

**Published:** 2024-01-01

**Authors:** Jinpiao Zhu, Chang Chen, Xiaodong Liu, Mengying He, Yuanyuan Fang, Li Wang, Junke Jia, Juan Guo, Ziyue Zhao, Chenyi Gao, Jingang He, Chengshi Xu, Fuqiang Xu, Daqing Ma, Jie Wang, Zongze Zhang

**Affiliations:** 1 Department of Anesthesiology, Department of Neurosurgery, Zhongnan Hospital, Wuhan University, Wuhan 430071, China.; 2 Department of Anesthesiology, The Children's Hospital, Zhejiang University School of Medicine, National Clinical Research Center for Child Health, Hangzhou, China.; 3 Department of Anesthesia and Intensive Care, Peter Hung Pain Research Institute, The Chinese University of Hong Kong, Hong Kong SAR, P.R. China.; 4 Key Laboratory of Magnetic Resonance in Biological Systems, State Key Laboratory of Magnetic Resonance and Atomic and Molecular Physics, National Center for Magnetic Resonance in Wuhan, Wuhan Institute of Physics and Mathematics, Innovation Academy for Precision Measurement Science and Technology, Chinese Academy of Sciences-Wuhan National Laboratory for Optoelectronics, Wuhan 430071, China.; 5 Department of Anesthesiology, Zhongshan Hospital, Fudan University, Shanghai, China.; 6 University of Chinese Academy of Sciences, Beijing 100049, China.; 7 Division of Anesthetics, Pain Medicine and Intensive Care, Department of Surgery and Cancer, Faculty of Medicine, Imperial College London, Chelsea and Westminster Hospital, London, UK.

**Keywords:** Consciousness, Cerebellum, Motor cortex, Neurotransmitters, Sevoflurane

## Abstract

**Background:** The neurobiological basis of gaining consciousness from unconscious state induced by anesthetics remains unknown. This study was designed to investigate the involvement of the cerebello-thalamus-motor cortical loop mediating consciousness transitions from the loss of consciousness (LOC) induced by an inhalational anesthetic sevoflurane in mice.

**Methods:** The neural tracing and fMRI together with opto**-**chemogenetic manipulation were used to investigate the potential link among cerebello-thalamus-motor cortical brain regions. The fiber photometry of calcium and neurotransmitters, including glutamate (Glu), γ-aminobutyric acid (GABA) and norepinephrine (NE), were monitored from the motor cortex (M1) and the 5^th^ lobule of the cerebellar vermis (5Cb) during unconsciousness induced by sevoflurane and gaining consciousness after sevoflurane exposure. Cerebellar Purkinje cells were optogenetically manipulated to investigate their influence on consciousness transitions during and after sevoflurane exposure.

**Results:** Activation of 5Cb Purkinje cells increased the Ca^2+^ flux in the M1 CaMKIIα^+^ neurons, but this increment was significantly reduced by inactivation of posterior and parafascicular thalamic nucleus**.** The 5Cb and M1 exhibited concerted calcium flux, and glutamate and GABA release during transitions from wakefulness, loss of consciousness, burst suppression to conscious recovery. Ca^2+^ flux and Glu release in the M1, but not in the 5Cb, showed a strong synchronization with the EEG burst suppression, particularly, in the gamma-band range. In contrast, the Glu, GABA and NE release and Ca^2+^ oscillations were coherent with the EEG gamma band activity only in the 5Cb during the pre-recovery of consciousness period. The optogenetic activation of Purkinje cells during burst suppression significantly facilitated emergence from anesthesia while the optogenetic inhibition prolonged the time to gaining consciousness.

**Conclusions:** Our data indicate that cerebellar neuronal communication integrated with motor cortex through thalamus promotes consciousness recovery from anesthesia which may likely serve as arousal regulation.

## Introduction

General anesthetics induce unconsciousness in humans, also namely, loss of righting reflex (LORR) in rodents but underlying mechanisms remain elusive. However, anesthetics were reported to disrupt the functional connectivity (FC) and the excitatory-inhibitory neurotransmitter systems within cerebral cortical network 1, 2, while restoration of FC and neurotransmitter transients within the sensory and motor cortical network is required to pass through the intermediate metastable states to be conscious after anesthesia 3, 4. The neurobiological characteristics of this recovery process, which involves the reintegration of consciousness and responsiveness after major network disruptions, are still largely unknown.

The reciprocal relationship with the high-order thalamus and cortex plays a vital role in modulating different states of consciousness 5, 6 Indeed, the strong association among thalamic and deep-layer cortical spiking activity and the level of consciousness was demonstrated to effectively awaken macaques from a state of stable anesthesia through stimulation to the thalamus 6. Interestingly, the cerebellum, one of the main sources sending output to the thalamus and further connecting with the primary motor cortex (M1) 7, can profoundly modulate cortical coherent activity 8. The cerebellum exhibits stage-dependent activity linked to wakefulness, and disruptions in this region can result in wakefulness disorders 9, 10. It is essential to conduct further research to elucidate whether the synchronization of neuronal activity between the cerebellum and the cortices represents an additional dimension of neuronal information integration between loss of consciousness (LOC) and recovery of consciousness (ROC) switch under general anesthesia conditions.

The synchronization of neuronal activity is considered to be essential for information processing in the nervous system. Coherent changes were to be task-specific and correlated with neural transmission effectiveness 11, 12. Both rapid neuronal excitation and inhibition in the cerebellum and cerebral cortex are mediated primarily by glutamate (Glu) and γ-aminobutyric acid (GABA) neurotransmission, while their disruption in cortical network is a key feature during anesthesia state 13, 14. Investigating the coherent changes of neuronal activity and their associated neurotransmitter release between M1 and the 5^th^ lobule of the cerebellar vermis (5Cb) of the cerebellum may provide further our understanding how specific brain regions coordinate during gaining consciousness from unconsciousness induced by general anesthetics.

This study was designed to investigate whether cerebellar 5Cb Purkinje cells and their communications with motor cortical M1 participate in gaining consciousness during anesthesia recovery through examining the coherent coordination between electroencephalogram (EEG) and neurotransmitter release/neuronal activity in the cerebello 5Cb -M1 loop in mice.

## Methods

### Animals

All animal procedures were carried out in compliance with National Institutes of Health animal care guidelines. Every effort has been made to minimize the total number of animals and their suffering. 9-week-old C57BL/6J mice were purchased from Liaoning Changsheng Biotechnology Co. (Liaoning, P.R. China), and the *Pcp2/L7-Cre* mice were generously provided by Prof. Ying Shen (Zhejiang University, Zhejiang, P.R. China). All animals were group-housed in the specific pathogen free (SPF) room with an ambient temperature of 23 ± 0.5 °C, a humility of 60% ± 2%, and an automatically controlled 12-h light-dark cycle (light on/off at 8:00/20:00). Mice had free access to food and water.

### Experimental paradigms

The whole experimental paradigms were illustrated in Figure S1. After virus injections and EEG/EMG electrode implantation, mice were allowed to recover for 3 weeks. Then the mice were placed in the anesthesia apparatus for 3 days for acclimatization. The apparatus consists of a transparent chamber (30 × 30 × 20 cm), which connects to a Fabius anesthesia machine (Dräger Co., Germany) and the Dräger monitor system (Germany) 15. A hole was drilled in the top of chamber for the recording wires to pass through. The animal body temperature was controlled by a heating pad and the physiological states were recorded using an animal monitoring system (PhysioSuite, Kent Scientific Co., USA). Anesthesia was induced and maintained by exposing to ~3% sevoflurane mixed with 60% O_2_, and the fiber photometry and EEG/EMG data were synchronously recoded.

### fMRI imaging

MRI data (n=8) were acquired using a 7.0-T small animal MRI scanner (Bruker Biospin GmbH, Germany) with a surface coil. The steps were similar with our previous study 16. The resting-state *f*MRI scanning data were acquired using the echo-planar imaging (EPI) sequence (repetition time (TR) of 1000 ms, echo time (TE) of 14 ms, flip angle of 45°, field of view (FOV) of 20 × 20 mm, and slice thickness of 1 mm). The T2 images were obtained as follows: TR = 5000 ms, effective TE = 12 ms, RARE factor = 8, matrix size = 256 × 256, and spatial resolution = 0.08× 0.08 mm.

The fMRI data were processed using various software, including AFNI 17, FSL 18 and ANTs 19. The EPI data were nonlinearly registered to the template, which was generated based on the T2 images of all animals. The T2 template was registered to the standard brain atlas template (ST) 20. The functional connectivity (FC) of the seed ROI-5Cb with the whole brain was analyzed according to the standard template of mouse brain. The degree centrality of each voxel was made to measure the voxel-wise FC strength 21. The differences in FC strength were analyzed by the permutation test.

### Virus injections

C57BL/6 and *Pcp2/L7-Cre* mice were anesthetized with ketamine/pentobarbitone. Their body temperature was maintained by a heating pad and the physiological states were monitored with the animal monitoring system (PhysioSuite, Kent Scientific Co., USA) throughout the virus injection procedure. The mouse head was stabilized in the stereotaxic instruments (RWD Life Science, Shenzhen, China). Lidocaine was then applied around the surgical area. The skull was exposed through a skin incision, and two small holes (1 mm in diameter) were drilled above the target brain sites (M1: AP +1.1mm, ML +1.5 mm, DV -1.5 mm; 5Cb: AP -6.0 mm, ML 0.0 mm, DV -1.5 mm). The glass micropipettes preloaded with viruses (200 nl) was connected to a pressure propelling apparatus (MICRO2T, World precision Instruments Co., Ltd) and gradually lowered into the brain regions to inject the virus (infusion speed =20 nl/min). AAV2/9-hSyn-GRABeen-NE1h-WPRE-pA (NE1h), AAV2/9-hSyn-iGABASnFR-WPRE-pA (GABA) and AAV2/9-hSyn-SF-iGluSnFR-WPRE-pA (Glu) (Brain Case, Shenzhen, China) were injected into the M1 and 5Cb regions of C57BL/6 mice. 6×10^5^ PFU H8 (150 nl) was injected into the 5Cb of C57BL/6 mice for neural circuit labeling. AAV2/9-CaMKIIα-GCaMP6f-WPRE-pA was injected into the M1, and AAV2/9-hEF1α-DIO-GCaMP6f -WPRE- pA into the 5Cb of *Pcp2/L7-Cre* mice. After the injection, the glass micropipette was kept in position for 10 minutes to avoid back diffusion.

### EEG/EMG electrodes implantation

As mentioned in previous studies 22-24, the EEG/EMG electrodes consisted of four stainless steel screws and two Teflon-coated silver leads. After the end of virus injection, two small holes were (1 mm in diameter) made above the frontal bone surface (AP +3.0 mm, ML ± 1.5 mm), and another two holes above the parietal bone surface (AP -1.5 mm, ML ± 1.5 mm). The EEG electrodes were implanted into these four bone holes and the two EMG electrodes were inserted into the bilateral trapezius muscle. The animals were recovered for three weeks before recording. The EEG/EMG electrodes were connected to a six-pin plastic plug electrode assembly, which was positioned on the skull with dental acrylic resin.

### Fiber photometry

After the virus injections, an optical fiber was positioned in a ceramic ferrule (230 μm in diameter, Xi 'an Bogao Optoelectronic Technology Co., LTD) and inserted into the regions of M1 and 5Cb for fiber photometry. The signals were collected by a laser beam through a 488 nm simulator (OBIS 488LS; Coherent), reflected off a dichroic mirror (MD498; Thorlabs), focused by an objective lens (Olympus, Japan), and coupled through a fiber collimation package (F240FC-A, Thorlabs) into a patch cable. The cable was connected to fibers chronically implanted in the mouse. Fluorescent signals were bandpass filtered (MF525-39, Thorlabs) and collected by a photomultiplier tube (CMOS, IDS imaging).

As described in previous studies 25, 26, the photometry data were imported to MATLAB R2020b MAT files for further analysis. The data was smoothed with a moving average filter and segmented based on behavioral events. The values of fluorescence change (*ΔF/F*) were calculated by* (F-F_0_)/F_0_*, where *F_0_* is the baseline fluorescence signal averaged over a 1.5 s control time window. Δ*F/F* values were illustrated by spectrogram or average plots with a shaded area indicating SEM.

### EEG/EMG analysis

EEG and EMG changes can serve as valuable indications for measuring transitions between unconscious and conscious state under general anesthesia 3, 27. In our study, we utilized the tethered data acquisition system (Medusa, Bio-Signal Technologies, China) to record EEG and EMG signals together with motor activity and behavioural changes of the mice to identify conscious (mixed frequency EEG signals and highly variable muscle tone) and unconscious (continuous slow waves and low EMG signals with high-amplitude sharp waves and/or isoelectric suppression) states 28, 29. The signals were amplified, filtered and digitalized with a resolution of 1000 Hz. Data were converted to European data format (edf) by Bio-utility software. The EEG/EMG signals were synchronized with the photometry data using the custom MATLAB code. Similar to the previous studies 30, the raw frequency data were transformed into an EEG spectrogram using the MATLAB. For the quantification of burst suppression ratio (BSR), a former calculation method was utilized, which was descried in previous studies 31, 32. Briefly, the burst and suppression regions were separated according to the amplitude of EEG voltage, and the voltage interval was limited to 0.5 s according to the suppression waves of each mouse. If the amplitude of the EEG was less than the interval threshold, the suppression event was sampled and assigned to value 1. Otherwise, signals above the interval threshold were determined as burst event with the assignment of 0. The BSR was presented as the percentage of suppression event in every minute.

### Optogenetic experiments

We used *Pcp2/L7-Cre* mice to selectively transduce Purkinje cells. AAV2/9-hSyn-DIO-ChrimsonR-mCherry (ChrimsonR), AAV2/9-hSyn-DIO-eNpHR3.0-mCherry (NpHR), or AAV2/9-hSyn-DIO-mCherry (mCherry) control virus (Brain Case, Shenzhen, China) were microinjected into the 5Cb. To monitor the response of the M1 neuron to cerebellar input, AAV9-CaMKIIα-GCaMP6f and AAV9-hSyn-iGluSnFR were injected into the left and right M1, respectively, while ChrimsonR and AAV9-hEF1α-DIO-GCaMP6f were simultaneously infused into the 5Cb. Optical fibers were inserted into the 5Cb or (and) M1 and anchored by EEG electrodes in the skull and EMG electrodes into the bilateral trapezius muscle.

To validate Purkinje cell activation by optogenetic manipulation, an optic fiber (AP -6.00 mm, ML 0.00 mm, DV -1.20 mm) equipped with an LED (594 nm, for optical stimulation) and an optic fiber (AP -5.30 mm, ML 0.00 mm, DV -2.00 mm, backwards 45 degree) connected to the fiber photometry recording system were placed over the 5Cb according to the indicated coordinators. Mice were gently placed in the anesthesia chamber. Optogenetic activation (594 nm, power=5 mW, pulse width=100 ms at 5 Hz) or inhibition (594 nm, power=5 mW, pulse width= 1 s at 1Hz) was performed for five cycles of light on for 5 s and then off for 30 s. In experiments to monitor changes in burst suppression ratio (BSR), photostimulation was performed 500 s after the onset of sevoflurane exposure and lasted for 120 s. To examine the effects of Purkinje cell activities on LOC and ROC, time to LOC and time to ROC were recorded during sevoflurane anesthesia with or without optogenetic manipulation of Purkinje cells. The optical stimulation was carried out in two 5-min durations, e.g. phase I 0-300 s and phase II 900-1200 s. Then the time to LOC and the time to ROC were determined based on EEG and EMG signals.

### Chemogenetic experiments

To validate whether 5Cb Purkinje cells regulate M1 neuron activity via thalamic pathways, we employed a series of viral injections. AAV2/9-hEF1a-DIO-hChR2-EGFP-WPRE-pA (AAV-DIO-ChR2) was injected into the 5Cb, AAV9-CaMKIIα-GCaMP6f (AAV-CaMKIIα-GCaMP) into the M1, and AAV2/9-hSyn-hM4D (Gi)-EGFP-ER2-WPRE-pA (AAV2/9-hSyn-hM4D (Gi) (Brain Case, Shenzhen, China) into the bilateral posterior thalamic nuclear group (Po) (AP -2.30 mm, ML ± 1.50 mm, DV -3.25 mm) or parafascicular thalamic nucleus (PF) (AP -2.30 mm, ML ± 0.75 mm, DV -3.25 mm) of the thalamus. Optical fibers were inserted into the 5Cb and M1. Following a 14-day recovery period, the mice underwent opto-chemogenetic experiments. Prior to the 30-minute photo-activation experiments, the mice received intraperitoneal injection of either saline or Clozapine-N-oxide (CNO, 2 mg/kg dissolved in saline; MedChemExpress). Optogenetic activation (470 nm, power=5mW, pulse width=100 ms at 5Hz) was carried out in five cycles of light-on for 1 second followed by light-off for 30 seconds. Finally, the Ca^2+^ activity of M1 CaMKIIα^+^ neurons was recorded using fiber photometry.

### Correlation of the EEG signal and neurotransmission/Ca^2+^ activity

The raw data of EEG spectrum were filtered at 0.3-1000 Hz using a custom program in MATLAB and further analysed using the MATLAB signal processing toolbox. The data was processed with a fast Fourier transform to calculate the absolute power spectrum of the frequency band every 0.02 min. Then the EEG activity was divided into different kinds of bands^33^, including delta (1-4Hz), theta (5-7Hz), alpha (8-12 Hz), beta (13-30 Hz) and Gama (31-90 Hz). Pearson correlation was used to assess the association between different EEG powers and neurotransmitter signal or neuronal activities 34, 35.

### Immunohistochemistry

Mice were anesthetized with isoflurane and then intracardially perfused with PBS and 4% PFA (Paraformaldehyde, Merck, Darmstadt, Germany) in PBS. The brain was removed and post-fixed in PFA for two days. The coronal brain sections (40 μm) were collected using a Thermo Fisher cryostat microtome (NX50, U.S.A) for the M1 and the whole brain with H8 injection. The sagittal cerebellum sections were collected for the 5Cb. To stain for CaMKIIα and D-28K, brain sections were washed three times with PBS and incubated with blocking solution for two hrs at room temperature. The sections were incubated with anti-CaMKIIα goat antibodies (1:500, ab87597, Abcam) or anti-D-28K mouse antibodies (1:500, C9848, Merck) at 4 °C for 48 hrs. Then the slices were washed with PBS for three times, and incubated with Cy3 rabbit anti-goat (1:500, 106831, Jackson) or Cy3 rabbit anti-mouse (1:500, 315-165-049, Jackson) for one hour at 37 °C. The slices for visualization of NE1h, GABA and Glu sensors were directly washed with PBS for three times. All slices were counterstained with DAPI (1:4000, C1002, Beyotime) before imaging on a microscope (Olympus VS120, Japan) using 10× and 40× objectives.

### Statistical Analysis

The sample size estimation was based on our pilot study of a significant increase of the Ca^2+^ activity of CaMKIIα^+^ neurons in the M1 from the baseline 1.36 (0.24) to 8.82 (2.62) following the opto-activation of Purkinje cells in the 5Cb. To achieve a desired power of 80% and with a type I error set at 0.05, a sample size of minimal 5 mice per group are needed. Therefore, 5-7 mice per group was used for subsequent experiments. Data were tested for normality using Shapiro-Wilk test, and were reported as mean [standard error of mean (SEM)]. Student's t-test was used to analyse differences with GraphPad Prism 8.0 (GraphPad Software, San Diego, CA, USA). A p value less than 0.05 was considered statistically significant.

## Results

### Structural and functional connectivity between cerebellum and motor cortex

**Figure 1:**
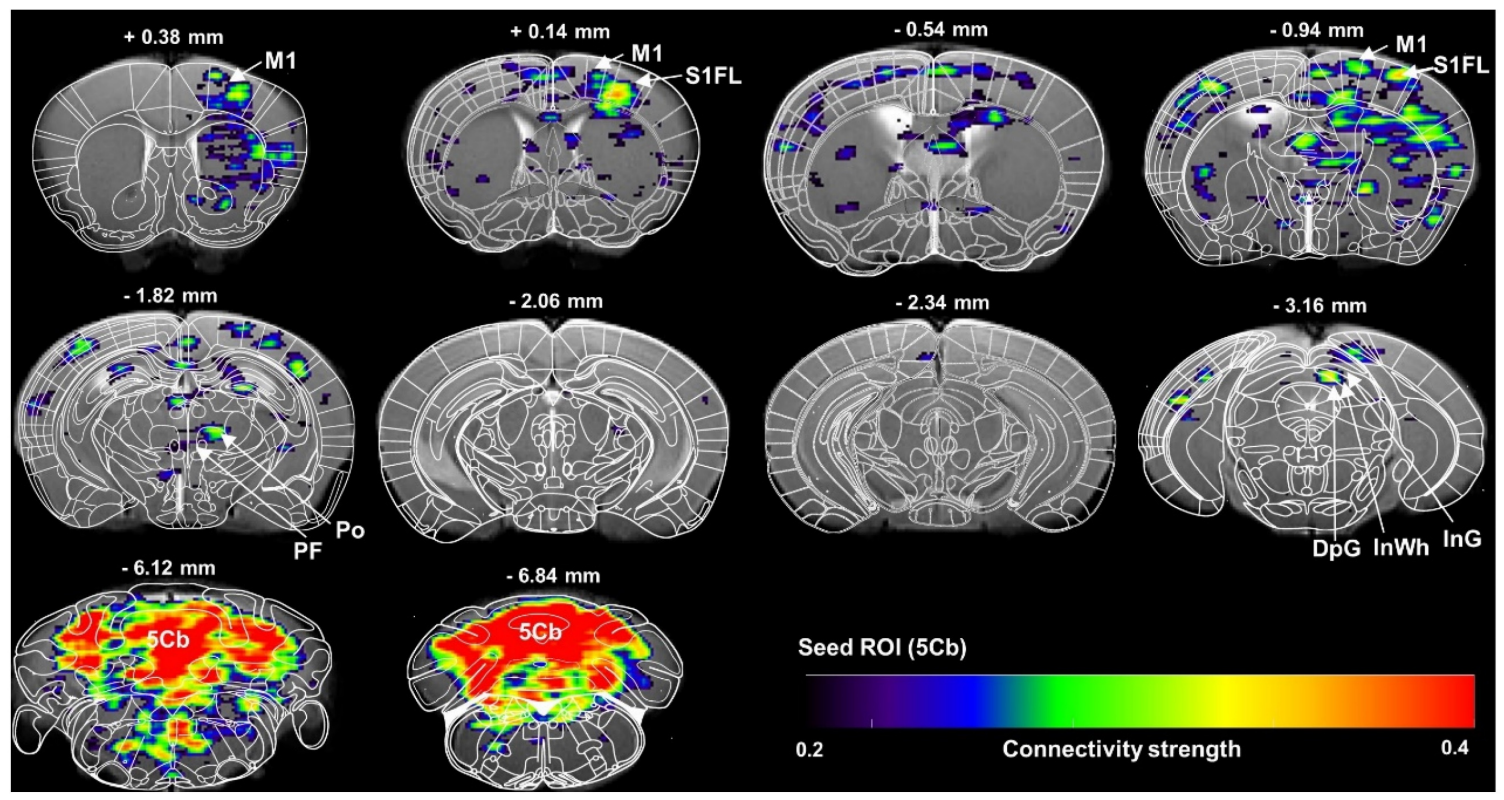
** Brain functional connectivity assessed with fMRI scanning of regional of interest (ROI) of the 5th lobule of the cerebellar vermis (5Cb) during anesthesia in mice.** The images were average from 7 mice. M1: primary motor cortex; S1FL: primary somatosensory cortex, forelimb region; Po: posterior thalamic nuclear group; PF: parafascicular thalamic nucleus; DpG: deep gray layer of the superior colliculus; InWh: intermediate white layer of the superior colliculus; InG: intermediate gray layer of the superior colliculus; 5Cb*:* the 5th lobule of the cerebellar vermis.

The cerebellum is critically involved in motor control 36, learning as well as cognitive process 37 and sleep/wake cycle 9. The outputs of the cerebellum to the cortical target or targets of the thalamic nuclei are not fully known. In this study, a high-brightness anterograde transneuronal herpes simplex virus (HSV, termed H8) tracer was injected in the 5^th^ lobule of the cerebellar vermis (5Cb) (Figure S2A-B). Five days after injection of H8 into 5Cb, the GFP-positive cells were found in the regions including intermediate geniculate nucleus (InG) of the superior colliculus (Figure S2C), posterior thalamic nuclear group (Po), parafascicular thalamic nucleus (PF) and ventral posteromedial thalamic nucleus (VPM) of thalamus (Figure S2D) as well as internal capsule (ic) (Figure S2E). In the cortices, the GFP-labeled neurons were noted in the primary motor cortex (M1) (Figure S2F) and secondary motor cortex (M2) (Figure S2G). Based on the anatomical connections between cerebellum and motor cortex, the functional connectivity between 5Cb and M1 through thalamus during inhaled anesthetic exposure was assessed with fMRI and there was a strong connection among 5Cb, Po or PF in the thalamus and M1 and M2 in cortices (Figure 1).

**Figure 2:**
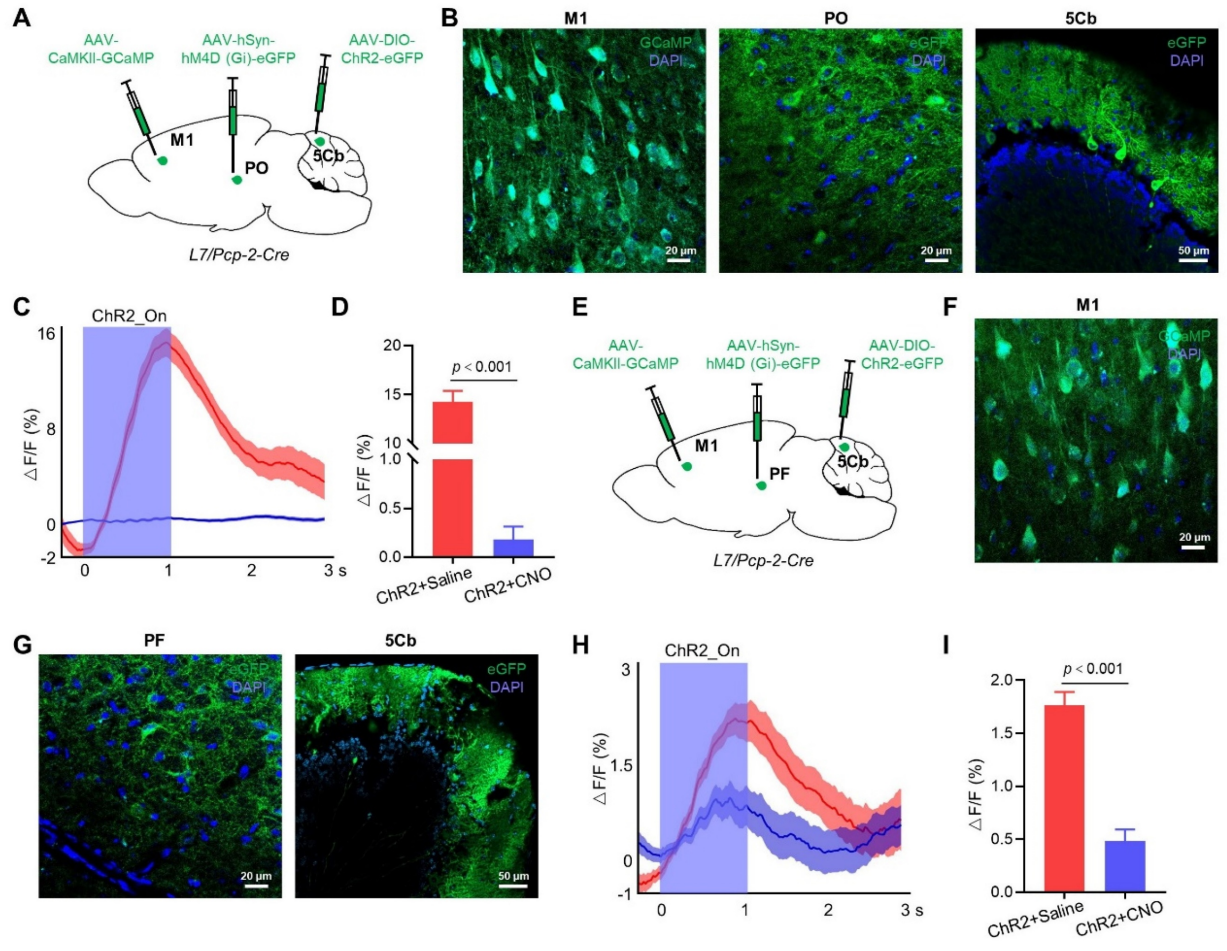
** Regulation of M1 cortical activity by 5Cb Purkinje cells via thalamus.** (A) Schematic representation of the virus injection sites in M1, Po and 5Cb of *Pcp2/L7-cre* mice. (B) Viral injection sites for GCaMP in M1, and eGFP in Po and 5Cb. (C) Averaged CaMKIIα^+^ neuron activity upon activation of Purkinje cells. Translucent red area indicates M1 CaMKIIα^+^ neuron activity in mice treated with saline (left, ChR2+Saline); translucent blue area indicates M1 CaMKIIα^+^ neuron activity in mice treated with CNO (right, ChR2+CNO). (D) Averaged ΔF/F values for ChR2+Saline and ChR2+CNO mice. (E) Schematic representation of the virus injection sites in M1, PF and 5Cb of *Pcp2/L7-cre* mice. (F-G) Viral injection sites for GCaMP in M1, and eGFP in PF and 5Cb. (H) Averaged CaMKIIα^+^ neuron activity upon activation of Purkinje cells. Translucent red area indicates M1 CaMKIIα^+^ neuron activity in ChR2+Saline mice; translucent blue area indicates M1 CaMKIIα+ neuron activity in ChR2+CNO mice. (I) Averaged ΔF/F values for ChR2+Saline and ChR2+CNO mice. Data are means ± SEM (n=5). M1: primary motor cortex; Po: posterior thalamic nuclear group; PF: parafascicular thalamic nucleus; 5Cb: the 5th lobule of the cerebellar vermis; CNO: Clozapine-N-oxide.

For a more in-depth exploration of whether the 5Cb regulates M1 cortical activity through the Po of the thalamus, we injected AAV-CaMKIIα-GCaMP into the M1 to monitor the CaMKIIα^+^ neuron activity during the optogenetic activation of the 5Cb Purkinje cells transfected with AAV-DIO-ChR2-eGFP in Pcp2/L7-Cre mice (Figure 2A-B). Activation of Purkinje cells increased the Ca^2+^ flux in the CaMKIIα^+^ neurons of the M1, but this increment was significantly reduced by the Po neuron inactivation (14.3±1.1 v.s. 0.18±0.13) (Figure 2C-D). We also tested the role of the PF neurons in the neural circuits between the 5Cb and the M1. AAV-hSyn-hM (Gi)-eGFP was bilaterally injected into the PF, along with AAV-CaMKIIα-GCaMP into the M1 and AAV-DIO-ChR2-eGFP into the 5Cb of the Pcp2/L7-Cre mice (Figure 2E-G). Inactivation of neurons in the PF reduced the increased Ca^2+^ activity (1.77±0.29 v.s. 0.48±0.25) in the CaMKIIα^+^ neurons upon activation by Purkinje cells, but the effect was weaker than that of the Po neuron inactivation (Figure 2H-I).

### Synchronous [Ca^2+^] activity in the M1 and 5Cb during transitions between unconsciousness and consciousness

**Figure 3:**
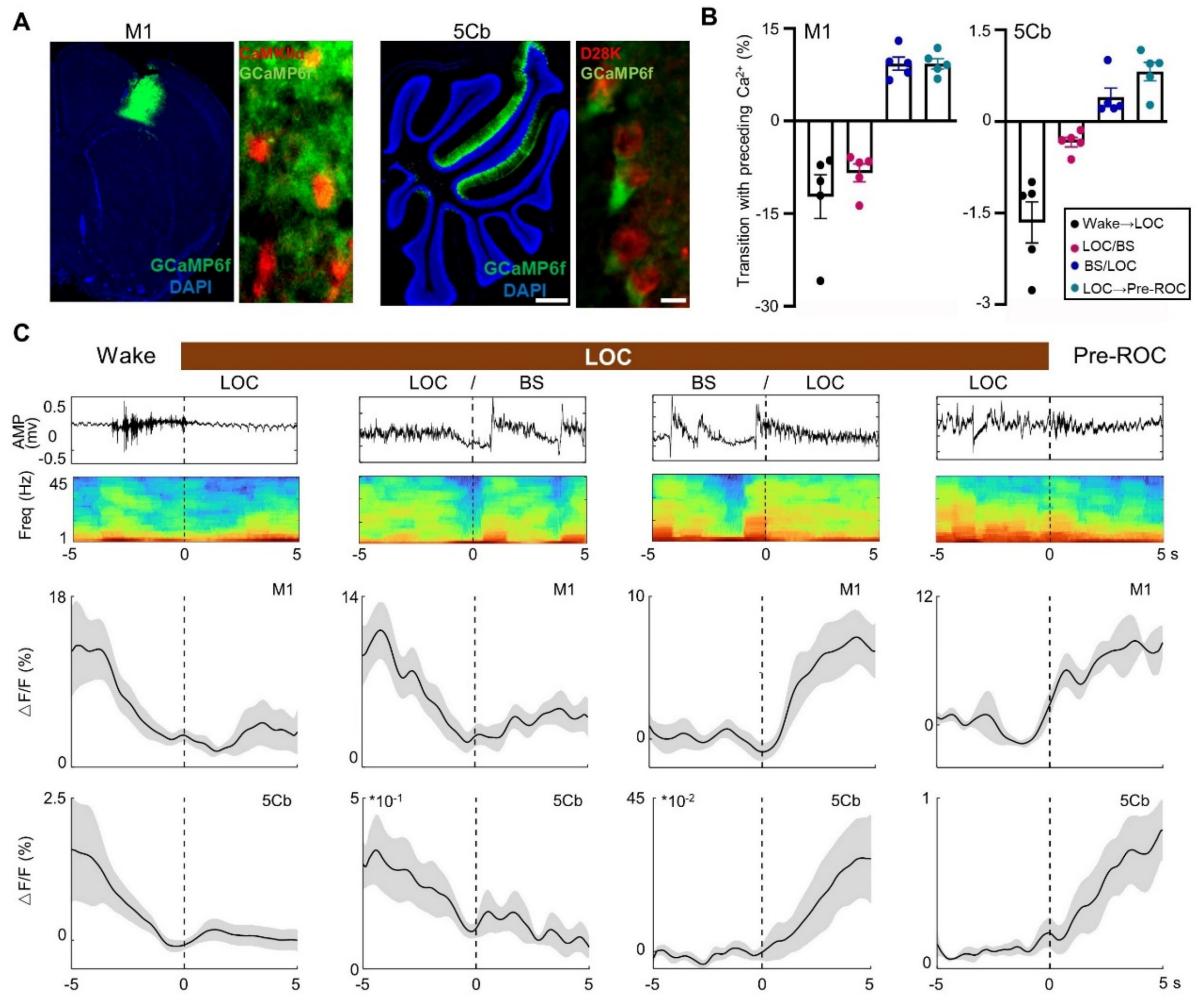
**Synchronous [Ca^2+^] activity in the primary cortex (M1) and the 5th lobule of the cerebellar vermis (5Cb) during different experimental course from wake, loss of consciousness to recovery of consciousness under 3% sevoflurane exposure in mice.** (A) Two viruses (AAV2/9-CaMKIIα-GCaMP6f-WPRE-pA and AV2/9-hEF1α-DIO-GCaMP6f-WPRE-pA were injected into the M1 and 5Cb in *Pcp2/L7-Cre* mice, respectively, and Ca^2+^ activities were determined with fiber-optic recording systems. Cortical CaMKIIα^+^ neurons were identified with CaMKIIα antibody (red) and Purkinje cells with D28K antibody (red); (B) The mean data of Ca^2+^ signals (Δ*F/F*) in the M1 and 5Cb between consciousness and unconsciousness transitions induced by sevoflurane. (C) Time-course of (top to bottom) EEG signals, EEG spectrum and variations of Ca^2+^ signals (Δ*F/F*) in the M1 and 5Cb from wake state towards loss of consciousness (LOC) to earlier recovery of consciousness (Pre-ROC). Data are means ± SEM (n=5). BS: EEG burst suppression.

The 5Cb Purkinje cells and M1 CaMKIIα^+^ neurons are highly activated during wakefulness and movement 38, 39. In this study, AAVs carrying CaMKIIα-GCaMP6f and Cre-dependent GCaMP6f were injected into the M1 and 5Cb of *Pcp2/L7-Cre* mice (Figure 3A), respectively. We then examined the simultaneous [Ca^2+^] activities with fiber photometry in the M1 CaMKIIα^+^ neurons and cerebellar Purkinje cells from wake state towards loss of consciousness (LOC) to earlier recovery of consciousness (Pre-ROC). The transition from wakefulness to LOC was accompanied by a concerted decrease in Ca^2+^ flux in the M1 and 5Cb (Figure 3B-C). During loss of consciousness accompanying with EEG burst suppression, a deep anesthesia-related EEG pattern 28, a further decrease of Ca^2+^ flux was found in both brain regions. In contrast, both M1 and 5Cb had a Ca^2+^ flux increment during burst suppression towards recovery of consciousness transitions (Figure 3B-C). These results suggested that both M1 CaMKIIα^+^ neurons and 5Cb Purkinje cells were likely activated concurrently to coordinate the cerebrocerebellar interactions in the unconscious and conscious states induced by general anesthetics. Given the fluctuating calcium flux during state transition, it is important to investigate the dynamic changes in neurotransmitters that might be associated with neuronal excitability.

### Neurotransmitters dynamics in the M1 and 5Cb between unconsciousness and consciousness transitions

**Figure 4:**
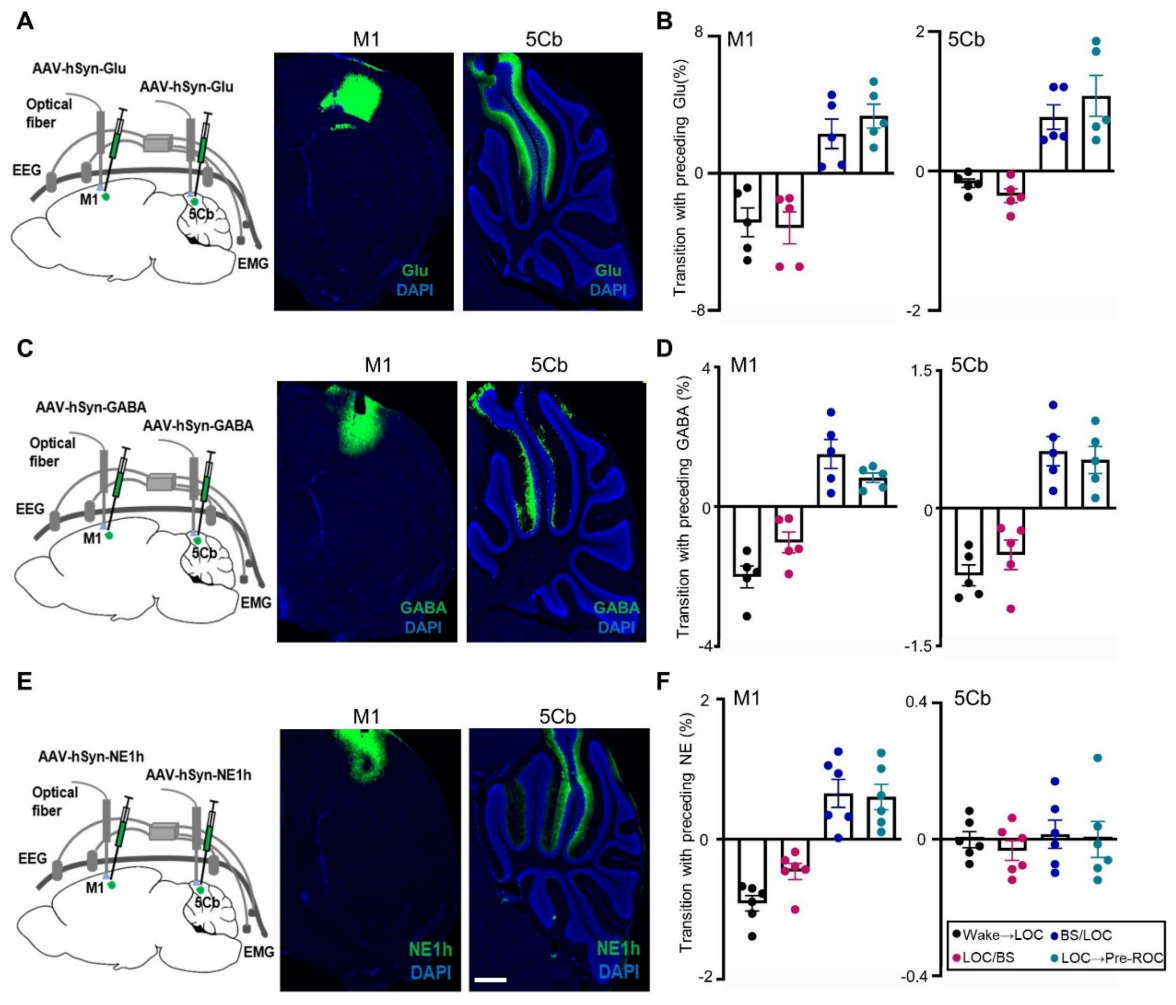
** Synchronous glutamate (Glu), γ-aminobutyric acid (GABA) and norepinephrine (NE) release in the primary cortex (M1) and the 5th lobule of the cerebellar vermis (5Cb) during different experimental course from wake, loss of consciousness to recovery of consciousness under 3% sevoflurane exposure in mice.** (A) AAV2/9-hSyn-SF-iGluSnFR-WPRE-pA (Glu) was injected into the M1 and 5Cb. (B) Glu release (Δ*F/F*) in the M1 and 5Cb from wake state towards loss of consciousness (LOC) to earlier recovery of consciousness (Pre-ROC). (C) AAV2/9-hSyn-iGABASnFR-WPRE-pA (GABA) was injected into the M1 and 5Cb. (D) GABA release (Δ*F/F*) in the M1 and 5Cb from wake state towards loss of consciousness (LOC) to earlier recovery of consciousness (Pre-ROC). (E) AAV2/9-hSyn-GRABeen-NE1h-WPRE-pA (NE) was injected into the M1 and 5Cb. (F) NE release (Δ*F/F*) in the M1 and 5Cb from wake state towards loss of consciousness (LOC) to earlier recovery of consciousness (Pre-ROC). Data are means ± SEM (n=5-6). BS: EEG burst suppression.

The transition between different states of brain activity is tightly regulated by the excitatory or inhibitory neurotransmitters. Glutamate (Glu) release was measured in our study and the fluorescent indicator iGluSnFR was transduced into M1 and 5Cb neurons (Figure 4A-B). The fluorescent intensity, which is proportional to the Glu levels, was monitored using fiber photometry through an implanted optical fiber. Glu fluorescent intensity were found to be simultaneously decreased in both M1 and 5Cb from wake state to EEG burst suppression (Figure 4A-B). In contrast, Glu release was increased observed in both brain regions during burst suppression towards recovery of consciousness transitions (Figure 4B). The similar pattern changes as Glu were found in GABA release measured with fiber photometry through GABA sensor (iGABASnFR) labelling (Figure 4C-D).

Norepinephrine (NE) is one of the major neurotransmitters in the regulation of consciousness states 40, 41. Several precious studies showed that anesthetics reduced NE release in locus coeruleus regions associated with sleep and wakefulness 42, 43. In this study, the extracellular NE dynamics in the M1 and 5Cb were simultaneously recorded and analyzed using the NE fluorescent indicator NE1h (Figure 4E). We found that there was a sustained reduction of the NE release from the wakefulness state to LOC and then LOC with EEG burst suppression transitions in the M1 (Figure 4F). This was followed by increased changes in the NE release during the LOC with EEG burst suppression and LOC towards ROC transitions in the same region (Figure 4F). In contrast, NE release in the 5Cb was not changed at all states (Figure 4F). Our data indicated that the fluctuations of NE in M1 were related to the anesthesia states but this was not in 5Cb (Figure 4F).

### Region-specific correlation of neurotransmitter/Ca^2+^ fluctuation and gamma-band EEG during unconsciousness with burst suppression

**Figure 5:**
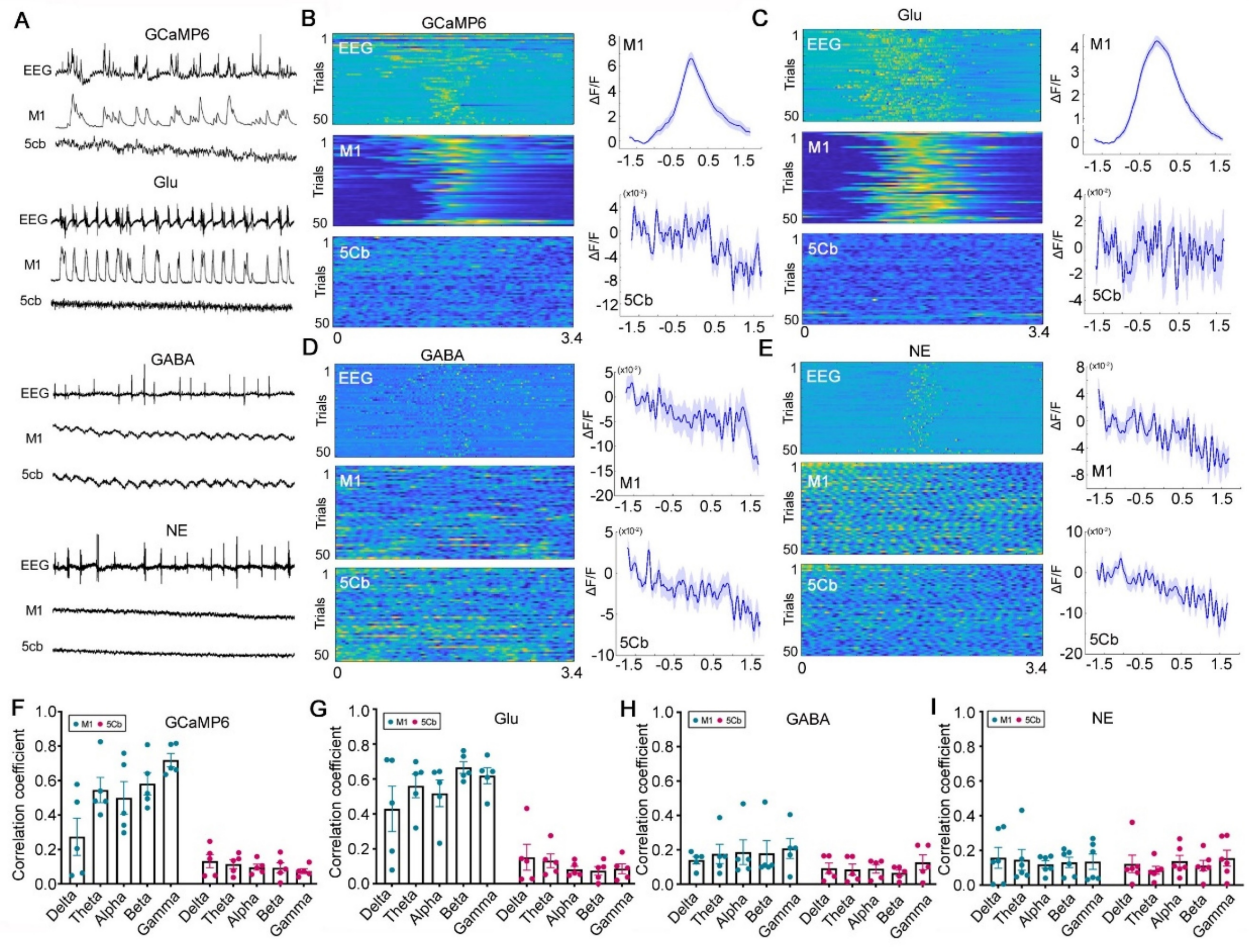
** [Ca^2+^] (GCaMP6) activity associated with glutamate (Glu), γ-aminobutyric acid (GABA) and norepinephrine (NE) release during EEG burst suppression (BS) induced by 3% sevoflurane exposure in the primary cortex (M1) and the 5th lobule of the cerebellar vermis (5Cb) in mice.** (A) Representative EEG changes paralleled with the fluorescence signals of GCaMP6 together with Glu, GABA and NE in the M1 and 5Cb during burst suppression; (B-E) GCaMP6, Glu, GABA and NE signals. Left panel, (upper) EEG spectrum and GCaMP6/Glu/GABA/NE signals in the M1 (middle) and 5Cb (bottom). Each row represents total data of 50 bouts collected. Right panel, the signal plots in the M1 (upper) and 5Cb (bottom). Blue lines indicate the mean values and shaded areas indicate SEM; (F-I) The correlation between the GCaMP6, GABA, NE release or Glu activity in the M1 (blue) and 5Cb (red) and EEG Bands (delta, theta, alpha, beta, and gamma). Data are means ± SEM (n = 5-6).

Burst suppression is a common EEG pattern during deep general anesthesia 28 and deep coma state after brain trauma or cardiac arrest 44. The burst suppression EEG hallmark was considered due to global anatomical or functional network connectivity disruptions 45. We, therefore, examined the neurotransmitter release and Ca^2+^ activity during unconsciousness with burst suppression in the cerebello-M1 loop. Interestingly, the Ca^2+^ flux and Glu release but not GABA and NE only in the M1 not 5Cb was synchronized with EEG burst suppression induced by sevoflurane (Figure 5A-B). We found that the Glu signal fluctuations in M1 but not in 5Cb were highly synchronized with the EEG-burst suppression (Figure 5A-C). No changes in GABA and NE release were observed in both brain regions during the EEG burst suppression period (Figure 5A and 5D-E). In addition, the correlations between the five frequency bands (delta, theta, alpha, beta, and gamma) of EEG signals and neurotransmitters/Ca^2+^ fluctuations in the M1 and 5Cb were further analyzed and the data showed that Ca^2+^ flux and Glu release significantly correlated with EEG oscillations in all frequency bands, in particular gamma band, in the M1 but not in the 5Cb (Figure 5F-G). Conversely, GABA and NE in both brain regions were not associated with any EEG bands (Figure 5H-I). These data provided evidence that M1 CaMKIIα^+^ neuronal excitation during EEG burst suppression were due to the glutamatergic neurotransmission.

### Neurotransmitter release and Ca^2+^ activity during early gaining consciousness after sevoflurane exposure

**Figure 6:**
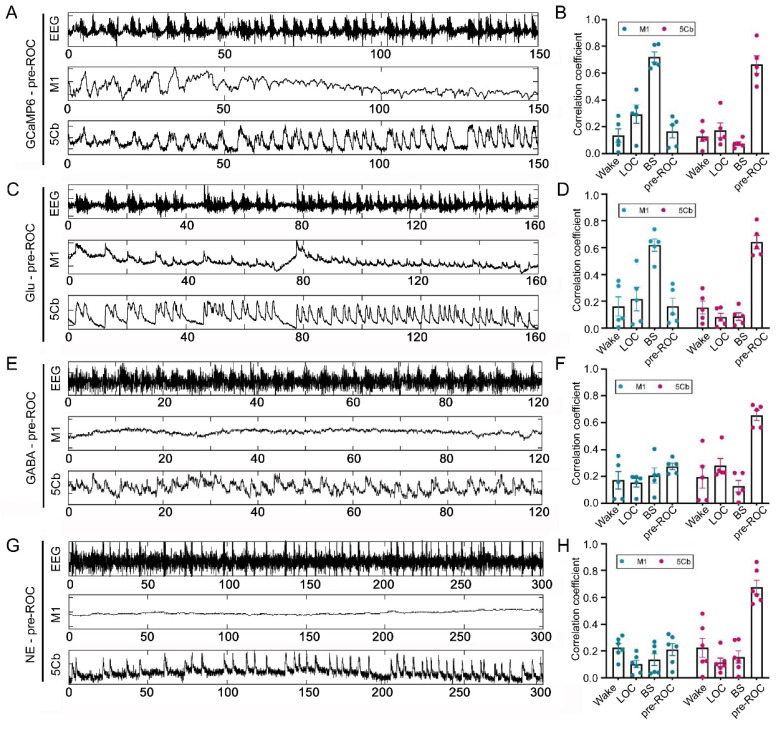
** Correlation of [Ca^2+^] (GCaMP6) activity, glutamate (Glu), γ-aminobutyric acid (GABA) /norepinephrine (NE) release and EEG gamma bands during earlier recovery of consciousness (Pre-ROC) after 3% sevoflurane exposure in the motor cortices (M1) and the 5th lobule of the cerebellar vermis (5Cb) in mice.** (A-B) EEG signals and GCaMP6 fluorescence intensities in the M1 and 5Cb during the Pre-ROC; The correlation between the GCaMP6 in the M1 (blue) and 5Cb (red) and EEG gamma band. (C-D) EEG signals and Glu fluorescence intensities in the M1 and 5Cb during the Pre-ROC; The correlation between Glu release in the M1 (blue) and 5Cb (red) and EEG gamma band. (E-F) EEG signals and GABA fluorescence intensities in the M1 and 5Cb during the Pre-ROC; The correlation between GABA release in the M1 (blue) and 5Cb (red) and EEG gamma band. (G-H) EEG signals and NE fluorescence intensities in the M1 and 5Cb during the Pre-ROC; The correlation between NE release in the M1 (blue) and 5Cb (red) and EEG gamma band. Data were means ± SEM (n = 5-6).

The EEG gamma-band oscillatory activity was closely related with the temporal dynamics of cortical networks 46. However, the association of neurotransmitter release/Ca^2+^ flux and EEG gamma-band together with responsible neurons in the cortical network during early gaining consciousness after sevoflurane exposure remains unknown. We found that EEG gamma-band oscillation was significantly correlated with Ca^2+^ flux in the M1 CaMKIIα^+^ neurons during unconsciousness with burst suppression induced by sevoflurane (Figure S3); However, the correlation in the 5Cb was only found during slowly gaining consciousness after sevoflurane exposure (Figure 6A-B). The correlation efficient between EEG gamma and Ca^2+^ flux in M1 (0.16) is lower than 5Cb (0.65). Surprisingly, the similar pattern was found for the correlation among all neurotransmitters (Glu, GABA and NE release) in the 5Cb and EEG gamma-band oscillation during earlier gaining consciousness (Figure 6C-H).

### Purkinje cells in the 5Cb promoted gaining consciousness after sevoflurane exposure

**Figure 7:**
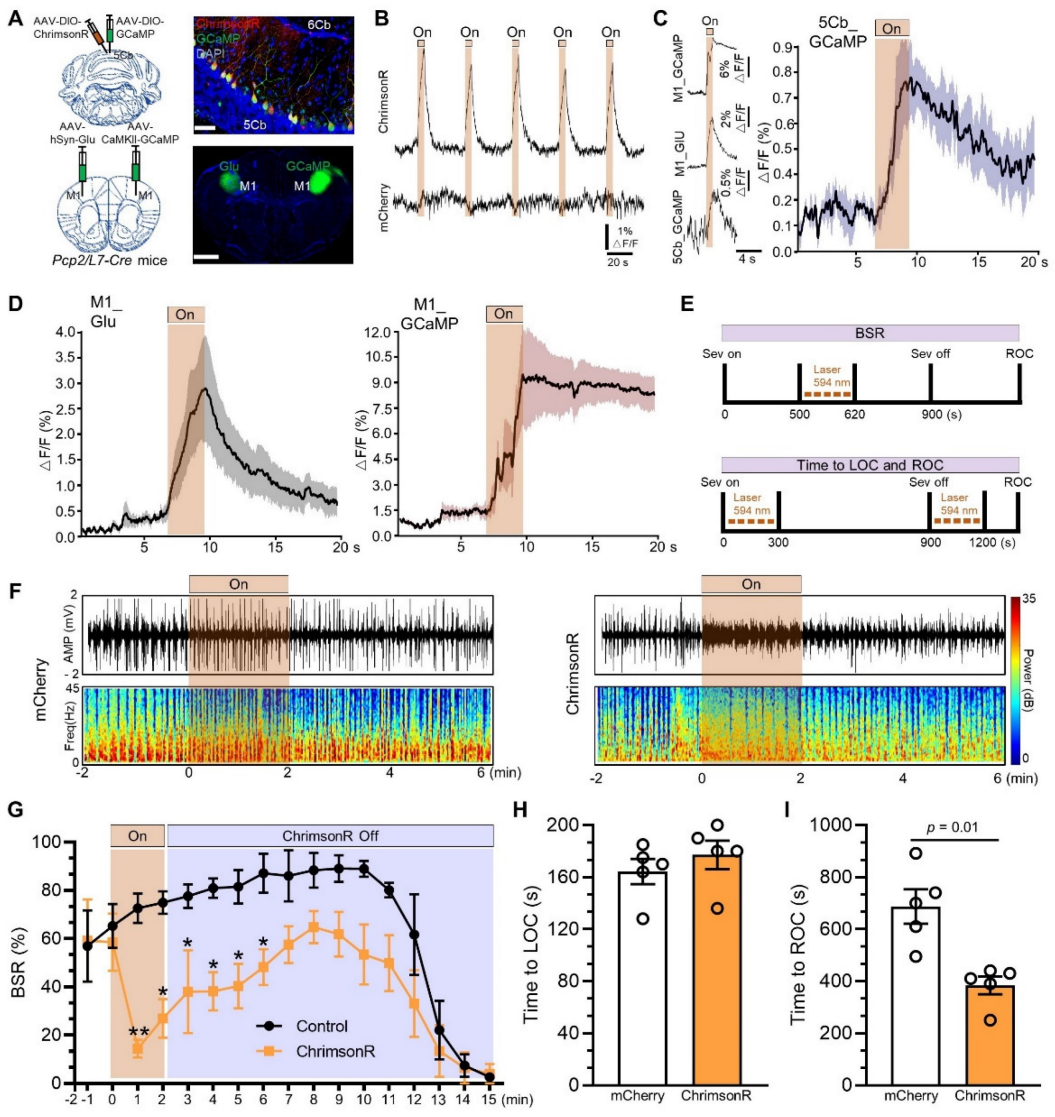
** Activated Purkinje cells in the 5th lobule of the cerebellar vermis (5Cb) promoted the recovery of consciousness after 3% sevoflurane exposure in mice.** (A) Viral-genetic manipulation for expressing ChrimsonR-mCherry (red) and GCaMP (green) in the 5Cb, and Glu (green) and GCaMP6 (green) in the left and right in the primary cortex (M1). (B) Purkinje cells activated with ChrimsonR (5s, 5Hz) in the 5Cb. The concerted changes among Purkinje cells of the 5Cb (C), and Glu and CaMKIIα^+^ neurons in the M1 (D) in response to Purkinje cells activated with ChrimsonR in the 5Cb. (E) Top panel: Optical stimulation during burst suppression; Bottom panel: Optical stimulation during loss of consciousness (LOC) and recovery of consciousness (ROC). (F) Representative EEG recordings and spectrum during and after opto-stimulation in the mCherry control and Purkinje cells activated with ChrimsonR. (G) The burst suppression ratio (BSR) per 1 min during 15 mins observation. (H, I) The time required to become LOC upon activation of Purkinje cells immediately after sevoflurane started (H) and the time needed to become ROC (I) upon activation of Purkinje cells immediately after sevoflurane termination. Data are mean ± SEM (n=5).

Next, we investigated the essential role of the 5Cb Purkinje cells in responding conscious recovery after sevoflurane exposure. *Pcp2/L7-Cre* mice were injected with AAV2/9-hSyn-DIO-ChrimsonR-mCherry (a red-shifted channelrhodopsin) and AAV2/9-hEF1α-DIO-GCaMP6f (Ca^2+^ indicator GCaMP6f) into the 5Cb to selectively activate the Purkinje cells in the 5Cb (Figure 7A, upper panel). Meanwhile, AAV2/9-hSyn-iGluSnFR (Glu release) and AAV2/9-CaMKIIα-GCaMP6f were injected into the left and right M1 (Figure 7A, bottom panel), respectively, for monitoring M1 neuronal activities after Purkinje cell activation. Photostimulation (100 ms pulse width at 5 Hz for 5 s) reliably evoked Ca^2+^ increases in the 5Cb Purkinje cells transfected with ChrimsonR but not with mCherry (Figure 7B). A rapid and concerted increase in Ca^2+^ flux and Glu release in the M1 CaMKIIα^+^ neurons upon photostimulation to the 5Cb (Figure 7C-D). Thus, the data presented here together with fMRI data (Figure 1) suggested a fast M1 neuronal activation modulated by signals from cerebellar output. In order to figure out the essential role of the 5Cb Purkinje cells in responding conscious recovery after sevoflurane exposure, Purkinje cell activation either during burst suppression (Figure 7E, upper panel) or during both sevoflurane exposure started and immediate after sevoflurane exposure (Figure 7E, bottom panel). Burst suppression was abolished and immediately switched to be EEG fast-wave activity when Purkinje cells were activated through ChrimsonR by photostimulation for 2 mins and this was not the case when Purkinje cells were not effectively activated via mCherry (Figure 7F). The burst suppression ratio (BSR) after Purkinje cell activation by photostimulation was decreased during 13 minutes of the observation period after the stimulation, in particular, it was significantly decreased by 80% during the first minute stimulation when compared to those in the mCherry controls (Figure 7G). Furthermore, the length from wake state to become unconsciousness induced by sevoflurane was not altered when Purkinje cells were activated at the beginning of sevoflurane exposure (Figure 7H) but the forced activation of Purkinje cells immediately after sevoflurane termination significantly shortened the time from unconsciousness to be wake state (Figure 7I).

**Figure 8:**
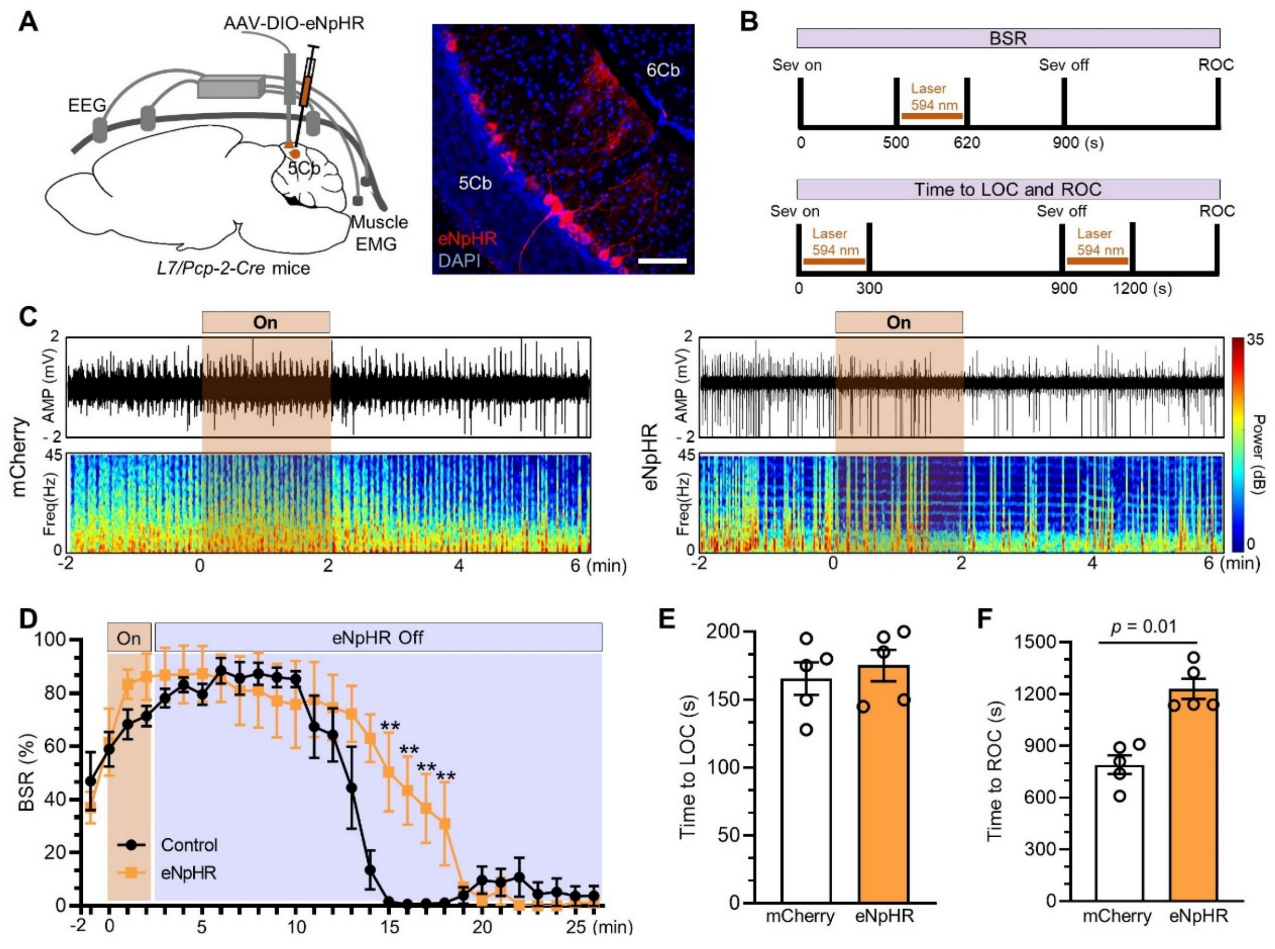
** Inhibited Purkinje cells in the 5th lobule of the cerebellar vermis (5Cb) prolonged the recovery of consciousness after 3% sevoflurane exposure in mice.** (A) Viral-genetic manipulation for expressing eNpHR-mCherry (red) in the 5Cb. (B) Top panel: Optical induced inhibition during burst suppression; Bottom panel: Optical induced inhibition during loss of consciousness (LOC) and recovery of consciousness (ROC). (C) Representative EEG recordings and spectrum during and after opto-inhibition in the mCherry control and Purkinje cells inhibited with eNpHR-mCherry. (D) The burst suppression ratio (BSR) per 1 min from burst suppression to wake state. (E, F) The time required to become LOC upon inhibition of Purkinje cells immediately after sevoflurane started (H) and the time needed to become ROC (I) upon inhibition of Purkinje cells immediately after sevoflurane termination. Data are mean ± SEM (n=5).

We next inhibited Purkinje cells with AAV2/9-DIO-eNpHR injected into the 5Cb of *Pcp2/L7-Cre* mice (Figure 8A) and the inhibition protocol as the cell activation described as above (Figure 8B). Burst suppression was slightly increased when Purkinje cells were inactivated through eNpHR by photostimulation for 2 minutes (Figure 8C, right panel) and it was not changed in the controls with mCherry (Figure 8C, left panel). The inhibition of Purkinje cells had no effect on the BSR during earlier phase (e.g. from minute 2 to minute 10 after stimulation) but significantly increased the BSR in the late phase (e.g. from minute 11 to minute 18 after stimulation) (Figure 8D). Interestingly, the length from wake state to become unconsciousness induced by sevoflurane was not changed when Purkinje cells were inhibited at the beginning of sevoflurane exposure (Figure 8E) but the forced inhibition of Purkinje cells immediately after sevoflurane termination significantly prolonged the time from unconsciousness to be wake state (Figure 8F).

## Discussion

**Figure 9:**
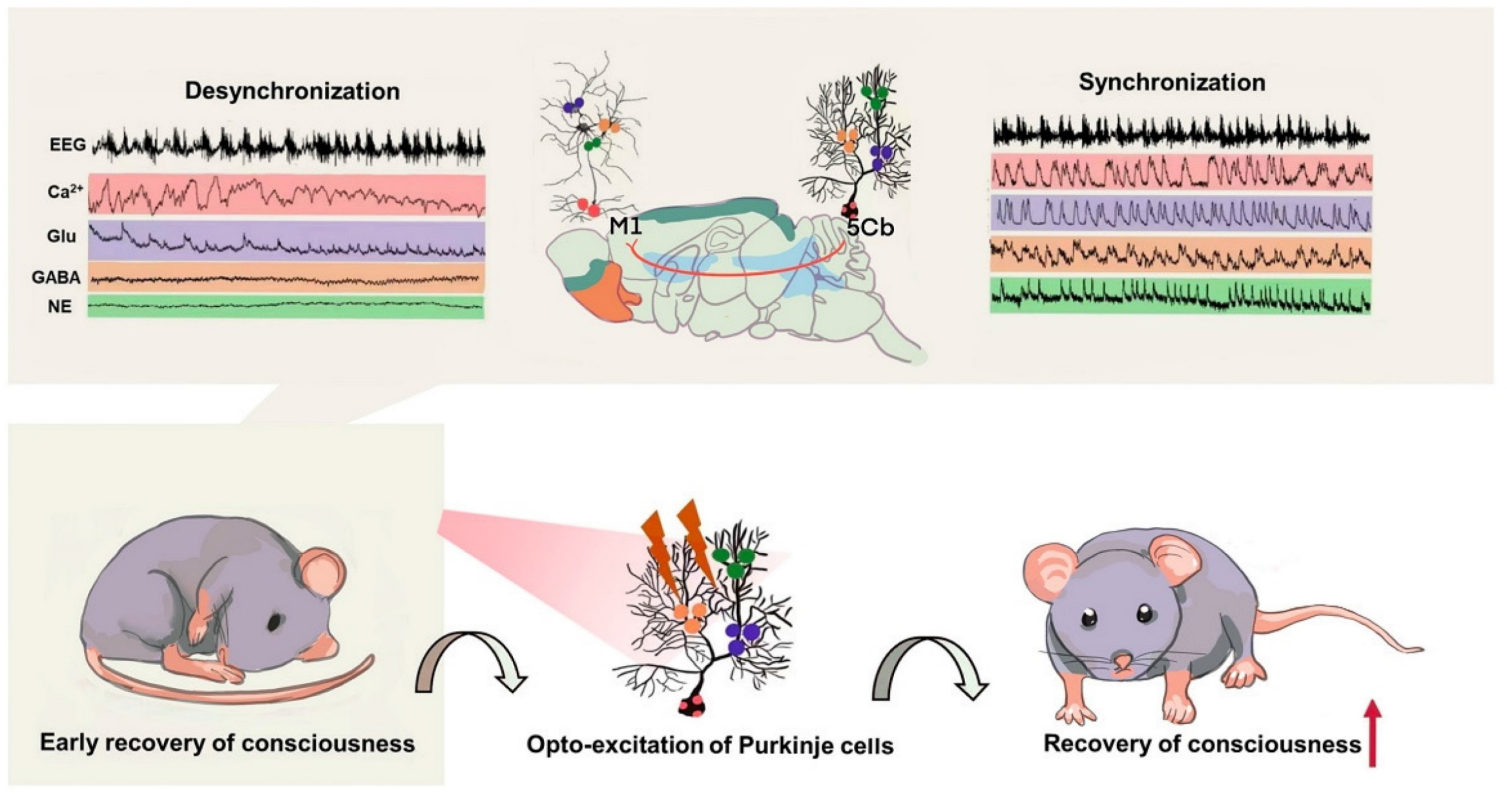
** Summary of the cerebellar-thalamus-motor cortex neural circuits promote gaining consciousness from unconsciousness induced by sevoflurane exposure. Top panel:** the synchronized changes of EEG associated with Ca^2+^ activities and neurotransmitters release including glutamate (Glu), γ-aminobutyric acid (GABA) and as well as norepinephrine (NE) in the 5th lobule of the cerebellar vermis (5Cb) but not the motor cortices (M1) during the early recovery of consciousness after sevoflurane withdrawal. **Bottom panel:** cerebellar Purkinje cell firing upon opto-stimulation during the early recovery of consciousness promotes conscious recovery from anesthesia.

Our current work indicated that the anatomical and functional connectivity among the cerebellum, thalamus and M1 cortex loop participated in conscious recovery after sevoflurane induced unconsciousness. Particularly, we found that the activated Purkinje cells in the 5th lobule of the cerebellar vermis (5Cb) promoted conscious recovery after sevoflurane exposure whilst their inhibition prolonged time to gaining consciousness after sevoflurane withdrawal. In line with previous studies 5, 47, our data further indicated that rapid transition from unconscious to conscious state requires feedback neuronal signals from Purkinje cells in the cerebellum 48, 49 and its co-ordination with the M1 cortex through thalamus (Figure 9).

In this study, we specifically found that Ca^2+^ flux in cerebellar Purkinje cells and M1 CaMKIIα^+^ neurons showed a strongly concerted changes accompanied by coherent and spontaneous Glu/GABA release in the both regions from the wakefulness state to loss of consciousness induced by sevoflurane. These changes were also seen from unconsciousness towards conscious recovery after sevoflurane withdrawal. During deep anesthesia as indicated with the EEG burst suppression, calcium oscillations and Glu release in the M1 cortex but not in the cerebellum were strongly correlated with the EEG gamma band activity. The release of GABA and NE did not showed synchronization with burst suppression pattern in either cortical M1 or cerebellar 5Cb. Instead, GABA and NE release was stationary during burst suppression suggesting that GABAergic and noradrenergic systems within the cerebellum-cortical network were not involved in the depth of anesthesia state. This was further supported by our data showing that the synchronicity of neuronal signals and EEG oscillation occurred only in the cortical M1 but not in 5Cb during the EEG burst period. Our study, therefore, indicate that the cerebellum is not involved in anesthetic-induced burst suppression but likely due to the subcortical network disruption by anesthetics such as sevoflurane. On the other hand, the synchrony of EEG bursts and Ca^2+^ signals in the cortical M1 showed that cortical-thalamic-cortical communications were preserved to some extent during the burst suppression phase and Ca^2+^ oscillation in the cortical M1 CaMKII^+^ neurons contributed to the switch between EEG burst and suppression shown in our study.

It has been reported that anesthetic-induced loss and recovery of consciousness was closely associated with the attenuation and augmentation of EEG gamma band activity 50, 51. Gamma oscillations were driven by a process of synchronized periodic inhibition generated either by GABAergic inhibitory interneurons or their interactions with excitatory glutamatergic neurons 52. Therefore, the synchronization and balance of excitatory and inhibitory neurotransmitters were closely related to the quality of wakeful consciousness during anabiosis 53, 54. We also found that a strong synchronization of neurotransmitter (Glu, GABA, and NE) release and Ca^2+^ flux with EEG gamma oscillations in the 5Cb during pre-awakening phase. Furthermore, a similar pattern of Ca^2+^ flux changes in cerebellar Purkinje cells and M1 CaMKIIα^+^ neurons which were accompanied by a concerted decrease and increase of Glu and GABA release in the cerebellum and M1 cortex, respectively, from wake towards loss of consciousness with or without EEG burst suppression induced by sevoflurane and then subsequent recovery of consciousness after sevoflurane withdrawal, suggesting that there is timely mutual communications between the cerebellum and M1 cortex during wake to unconsciousness transition. In addition, calcium spiking in cerebellar Purkinje cells together with their Glu, GABA and NE synchronized with EEG gamma band oscillation during early recovery of consciousness (Figure 6). All these suggest that cerebellum is preferentially involved in regulating and coordinating neuronal communications in the early stage of arousal. Indeed, our study suggests that long-range feedback projections from cerebellum to cortex are vital for consciousness and then cerebellum stimulates motor cortex via modulation of cross-regional, laminar-specific activity through involving gamma oscillations as found in our study.

Furthermore, previous studies suggested that synchrony of the EEG gamma range was also associated with the merging of information within thalamocortical networks 6, 55. It is well known that thalamus received powerful inputs from the cerebellum 7. Reactivation of the central lateral thalamic nucleus and deep cortical layer loop with gamma-frequency stimulation reinstated wake-like cortical dynamics and increased conscious level 6; This was further extended by our work showing that the presence of cerebellar-thalamic-motor cortex circuits contributed to conscious recovery from anesthesia. Our work reported here together with previous studies 9, 56 indicated that the integration and synchronization of neuronal communications along cerebello-thalamo-cortical pathway are very likely essential for arousal.

Cerebellum was documented to differentially modulate between sensory and motor cortex in EEG theta and gamma band activity 8. The networks and connections of cortico-pontocerebellar afferent and cerebello-thalamo-cortical efferent pathways were highly regulated and integrated to the cortical cerebral regions related to cognition and consciousness 56, 57. We found that optogenetic activation of cerebellar Purkinje cells triggered synchronized excitation of CaMKIIα^+^ neurons and glutamate release in the M1 and promoted recovery consciousness from EEG burst suppression. Conversely, optogenetic inhibition prolonged this recovery. Previous studies also reported that the cerebellum participated in fine-tuning and regulation of the sleep-wake cycle in a sleep-related cerebellum-dependent manner 9, 58. Taken together, our study for the first time showed that the cerebellar-thalamus-cortical M1 communication contributed to recovery of consciousness during anesthesia recovery.

The cerebellum has been identified as an essential coordinator of task-specific neuronal communication between multiple cerebral cortices *via* the modulation of coherent neuronal oscillations for wakefulness 8. Brain damage involved with cerebellum injury in coma patients has poor outcomes including more difficult gaining consciousness and death in particular for those who had EEG burst suppression 10. This is likely due to that damage to the cerebellum resulted in coordination of brain network lost, and therefore, jeopardized wakefulness in coma patients. Furthermore, converging evidence also suggested that structural coupling and synchronization arise in the recovery of motor and consciousness of coma patients 5, 6. Interestingly, we had a 57-year-old male patient who underwent the right acoustic neuroma resection under general anesthesia. Although the depth of anesthesia was adequate, his EEG BIS reading was timely increased from 40 to 93, indicating that the strong anatomical and functional connectivity between near cerebellum (surgical manipulation) and cortex (BIS recording) (Figure S4). The dissociation of consciousness and motor function was found up to 15-20% of coma patients in the intensive care unit who had poor 1-year outcomes 59. Our and previous work together with clinical observations discussed above may suggest that only “intact” cortical circuits without input from cerebellum may not be sufficient to maintain arouse. It is suggested that recovery of consciousness after anesthesia were involved in transmission reconnection and even reconstruction in the cerebral cortical circuits and the higher-order thalamocortical information integration 5, 6. In addition, neuropsychiatric complications such as agitation 60 or delirium 61 following anesthesia and surgery are common in elderly. The functional connectivity impairment between brain regions has been demonstrated in patients following cardiac surgery 62 but it is remaining unknown whether the cerebellar 5Cb-thalamus-cortical M1 loop may be also affected in those patients warrants further study.

## Conclusions

In this study, we found that the involvement of the cerebellar 5Cb- cortical M1 loop in different conscious states is highlighted by the concerted neurotransmitter release and neuronal activities during sevoflurane-induced unconsciousness transitions rather than depth of anesthesia. In addition, the synchronization of cerebellar neurotransmitter release/calcium oscillation and EEG gamma band activity during pre-consciousness recovery to gaining full consciousness indicates that cerebellar participation might be essential for speedy recovery of consciousness after anesthesia. The current work may also suggest that cerebellum health as a conscious promotor and potential predictor of the recovery under various pathophysiological conditions including coma, delirium and agitation.

## Supplementary Material

Supplementary figures.
