# Supplementary material for: Cerebellar Purkinje cell firing promotes conscious recovery from anesthesia state through coordinating neuronal communications with motor cortex
Source: Theranostics. 2024 Jan 1;14(2):480–95. doi: 10.7150/thno.89592 (PMC10758059; doi:10.7150/thno.89592)
Supplement: Supplementary file 1 — Supplementary figures. [file thnov14p0480s1.pdf]

Zhu J. et al. Cerebellar Purkinje cell firing promotes conscious recovery from anesthesia state through coordinating neuronal communications with motor cortex

Supplemental Figures

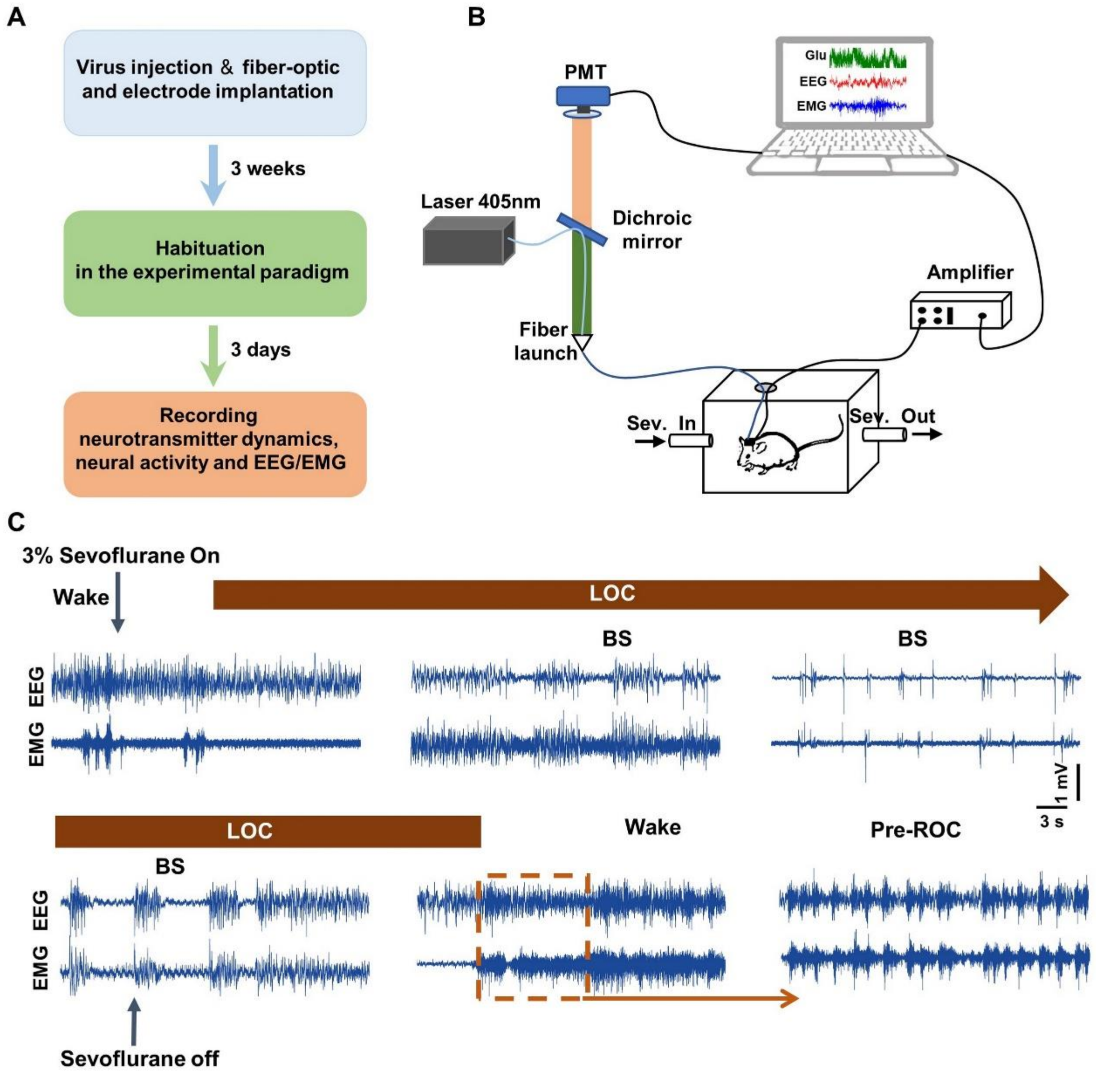

Figure S1. Experimental protocol, setup and EEG/EMG recordings under sevoflurane administration in mice. (A) Timeline of the whole experimental procedure, including virus injection, habituation, and recording procedures. (B) Illustration of the whole experimental setup. (C) EEG/EMG recording examples from one mouse from wake state towards loss of consciousness (LOC) to earlier recovery of consciousness (Pre-ROC). BS = EEG burst suppression.

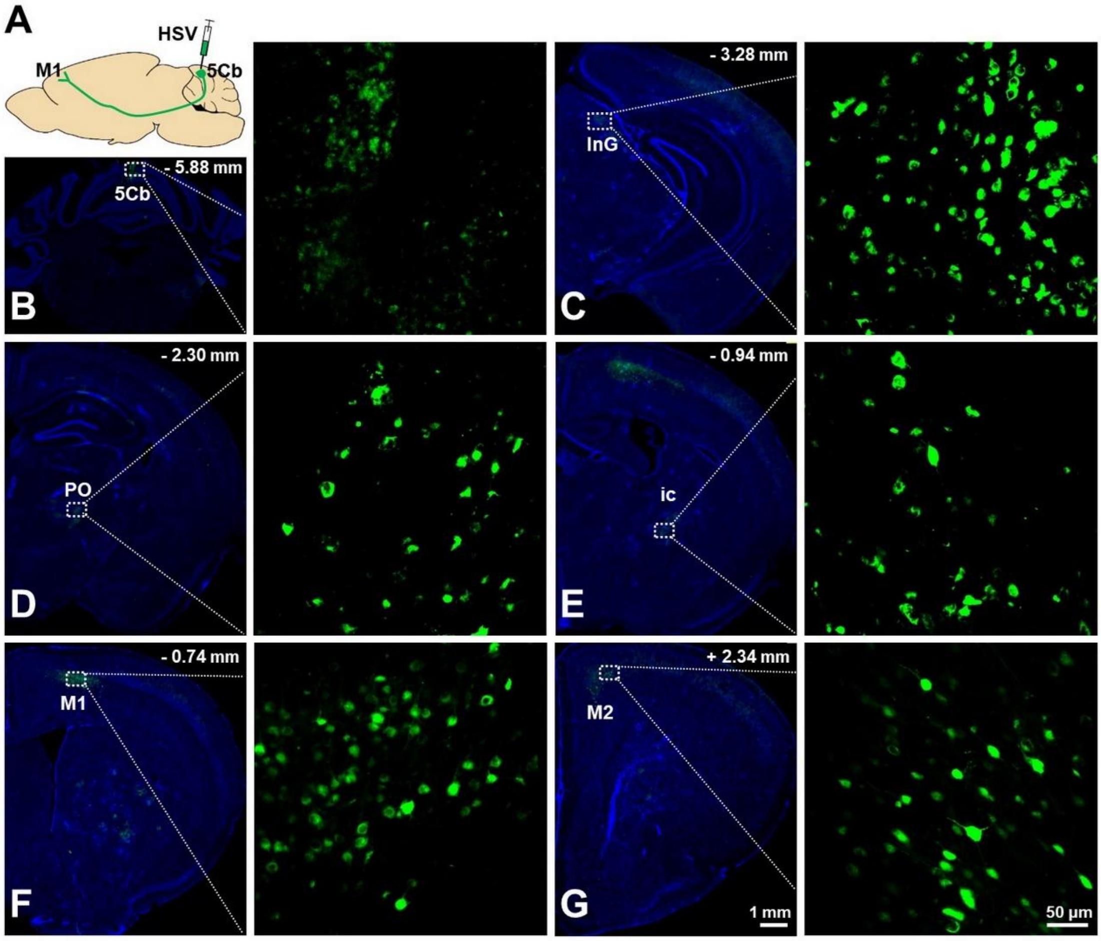

Figure S2. Mapping the cerebellum output pathway with HSV injection. (A) Illustration of HSV injection and tracing pathway. (B) The 5th lobule of the cerebellar vermis (5Cb) injection site. Selected targets are: (C) intermediate geniculate nucleus (InG) of the superior colliculus; (D) posterior thalamic nuclear group (Po) of thalamus (THA); (E) internal capsule (ic); (F) primary motor cortex (M1) and (G) secondary motor cortex (M2).

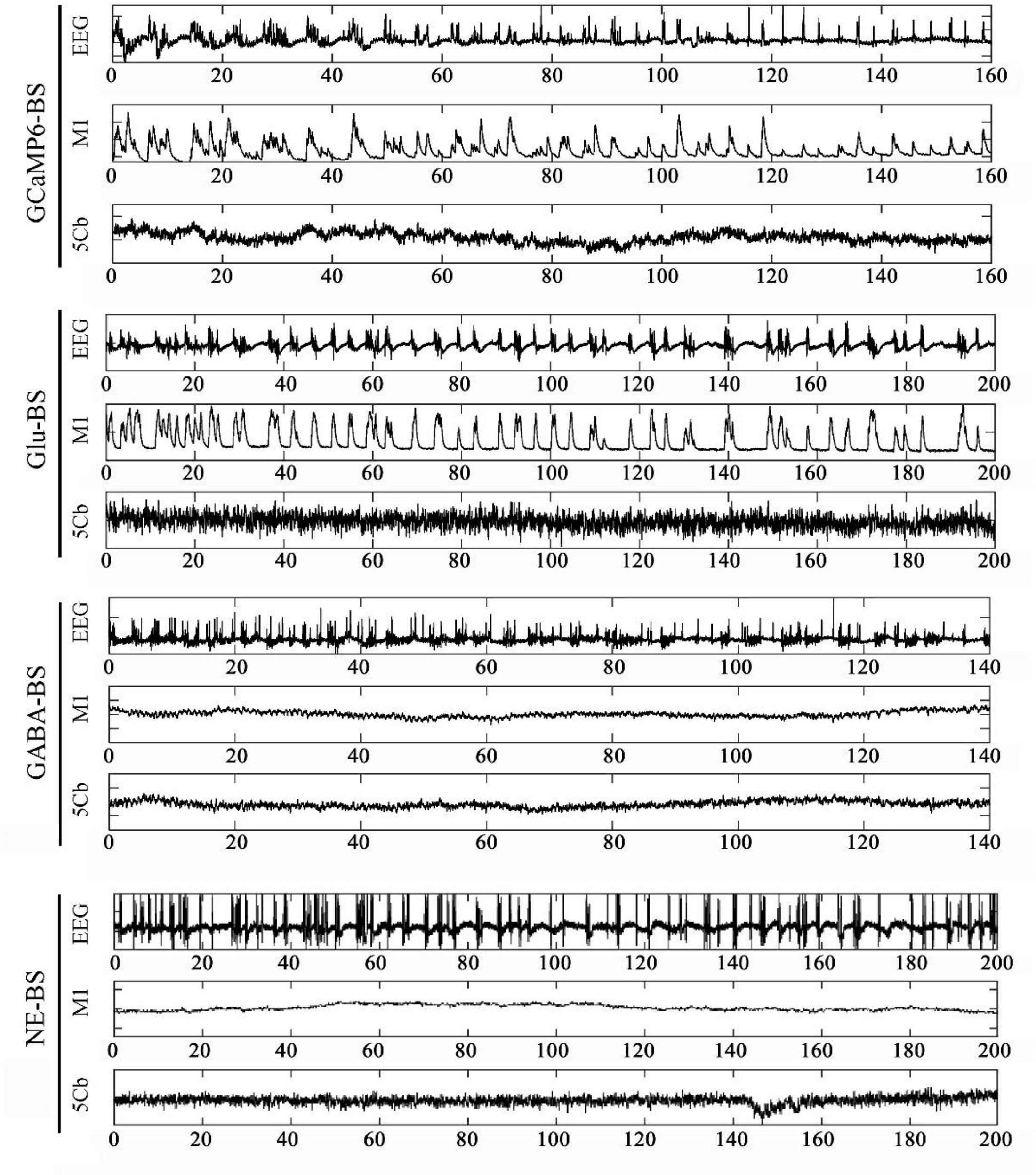

Figure S3. EEG signals and GCaMP6, Glu, GABA or NE release signals in the motor cortices (M1) and the 5th lobule of the cerebellar vermis (5Cb) during burst suppression.

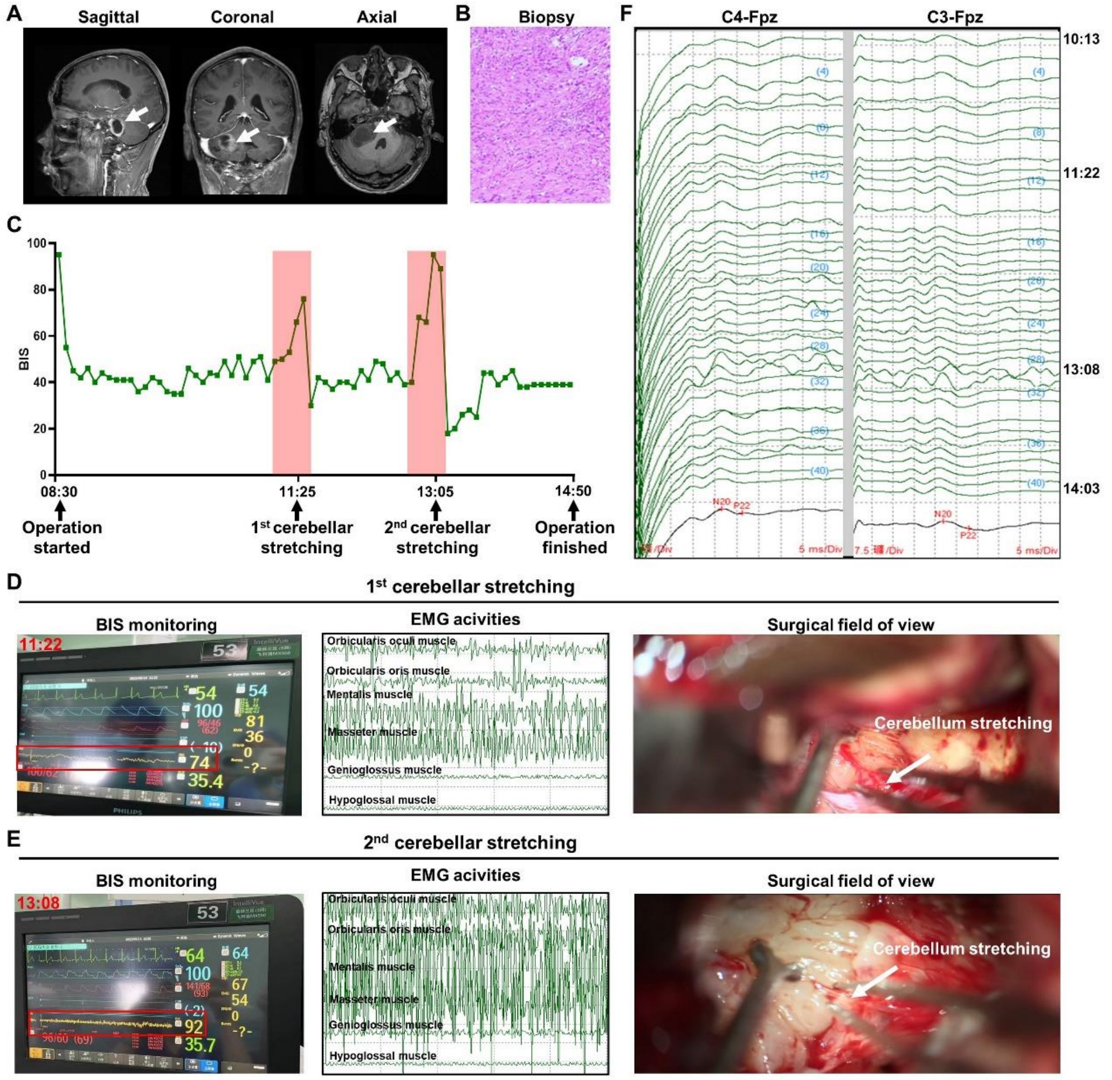

Figure S4. The EEG biospectral index (BIS) changes of EEG undergoing the right acoustic neuroma resection under general anestheesia in a 57-year-old male patient. The patient was anestheetised with sevoflurane, propofol, remifentanil hydrochloride and remazolam to keep the biospectral index (BIS, an useful tool for assessment the sedative-hypnotic state) between 40 and 60. Incidentally, the BIS reading was increased from 40 to 93 with EEG transition from slow-wave activity to fast-wave activity during surgical manipulation. The depth of anesthesia was adequate and these BIS changes indicated that the strong anatomical and functional connectivity between near cerebellum (surgical stimulastion) and cortex (BIS recording). (A) MRI image of the right acoustic neuroma; (B) Biopsy (hematoxylin and eosin stain) with schwannoma demonstrating cell histology. (C) The changes of BIS during the process of operation; (D) The response of the BIS (left panel) and the electromyography (middle panel), and the surgical field of view (right panel) during the 1st cerebellar stretching; (E) The response of the BIS (left panel) and the electromyography (middle panel), and the surgical field of view

(right panel) during the 2nd cerebellar stretching; (F) The electroencephalogram activity of the C4-Fpz and C4-Fpz during the process of operation.
